# Where have all the parasites gone? Unusual *Plasmodium falciparum* monoparasitaemia in a cross-sectional malariometric survey in northern Nigeria

**DOI:** 10.12688/f1000research.20997.2

**Published:** 2020-10-27

**Authors:** Usman Nasir Nakakana, Ben O. Onankpa, Ismaila Ahmed Mohammed, Ridwan M. Jega, Nma Muhammad Jiya

**Affiliations:** 1 Department of Paediatrics, Usmanu Danfodiyo University Teaching Hospital, Sokoto, Nigeria; 2 Medical Research Council Unit The Gambia at London School of Tropical Medicine and Hygiene, Fajara, The Gambia; 3 Department of Community Medicine, Usmanu Danfodiyo University Teaching Hospital, Sokoto, Nigeria

**Keywords:** Malaria, Nigeria, Plasmodium falciparum, PfPR2-10

## Abstract

**Background:** Malaria is caused by one of five currently known *Plasmodium* parasite species causing disease in humans. While modelling has provided information of the vector, the same is not entirely the case for the parasite. The World Malaria reports of 2014 to 2016 reported 100% of confirmed cases from Nigeria being due to *Plasmodium falciparum*. Generally, about 98% of cases of uncomplicated malaria in most regions surveyed in Nigeria recently is due to *P. falciparum*, with the remainder being due to *P. malariae*. This study aimed to determine the proportions of *Plasmodium* parasites causing uncomplicated malaria in Wamakko Local Government Area of Sokoto State, north-western Nigeria.

**Methods:** The study was a descriptive, cross-sectional study conducted during the rainy season and dry season in north-western Nigeria. The area has a ‘local steppe’ climate and Sudanian Savannah vegetation. Sampling was via multistage cluster sampling. Selected participants were examined for pallor, palpable splenomegaly and signs of complicated malaria. Blood samples were also taken for rapid diagnosis of malaria and thick and thin films to identify parasitaemia and the parasite species. Participants found to have malaria were treated with Artemether/Lumefantrine and those with complicated malaria were referred to the nearest hospital.

** Results:** We found a parasite prevalence of 34.8% overall, which was higher in the rainy season (49.3%) than in the dry season (20.2%). There was monoparasitaemia of *Plasmodium falciparum* throughout the study area, irrespective of the clinical status of the participant. Mapping of the parasite was extended throughout the Local Government Area and the State.

**Conclusions:** Despite the intermediate endemicity in the area. *P. falciparum* monoparasitaemia affirms theories of disappearance of other parasite species, either due to faltering control of *P. falciparum* or more efficient control of other species.

## Introduction

Malaria is caused by one of five currently known *Plasmodium* species causing diseases in humans. These are *P. falciparum, P. vivax, P. ovale, P. malariae* and *P. knowlesi.* Scientists have modelled the anopheles mosquito vector extensively based on its characteristics, but not for the parasite, it has been difficult to predict its preponderance and distribution, with notable exceptions ^1^. An absence of the Duffy antigen on red blood cells of West Africans has long been postulated to be responsible for the absence of *P. vivax* in these areas ^2^, but cases of *P. falciparum*, *P. malariae* and *P. ovale*, in order of decreasing occurrence, have been found. The World Malaria Reports of 2014 ^3^ and 2015 ^4^ reported 100% of confirmed cases in Nigeria as being due to *P. falciparum.* It is generally thought to account for about 98% of all malaria cases, with *P. malariae* accounting for the rest, often as a co-infection with *P. falciparum* ^5^. This figure likely over-estimates the proportion of cases as *P. falciparum* is responsible for most severe cases of malaria; these are the cases most commonly reported alongside confirmed cases of malaria, which are tracked by passive surveillance in Nigeria. It may also be because of limited expertise in identifying other species of *Plasmodium*.

Available data from across Nigeria shows a mixed picture; large areas across northern Nigeria in 1967/68 found an average proportion of 22% of malaria parasitaemia due to *P. malariae* in a study at a time when most of Nigeria was considered holoendemic for malaria. *P. malariae* was often seen in coinfection with *P. falciparum*, ** particularly among younger age groups. *P. ovale* was responsible for 5% of malaria infections, being more common in children under five years of age, and *P.falciparum* ranged between 84.4% and 90.5% across age groups ^6^. The proportion of *P. malariae* was high, probably because the data was obtained from a survey of both asymptomatic and symptomatic participants.

More recently, in 2010, prior to the commencement of nationwide LLIN distribution, a study including 4209 individuals in Jos, northern Nigeria, found a *P. malariae* rate as low as 1.6%, with *P. falciparum* responsible for 98.7% of infections, sometimes in co-infection with *P. malariae* ^5^. Children aged less than 10 years and all individuals in every third household were selected for this abridged malaria indicator survey (MIS). Crucially, however, no *P. vivax or P. ovale* species were seen either in this location or another in south-eastern Nigeria, which was shown to have a higher rate of *P. malariae* infections (around 30%) and lower rate of *P. falciparum* infections (68.1%) in a study also conducted in 2010 ^7^. This study, however, included 2,936 individuals from 1400 clusters in Abia state, spread out across the state. Quality control measures in the identification of parasite species were implemented in the study, including a WHO-certified malaria microscopist, giving credibility to the results obtained ^7^. In south-western Nigeria, a study in Ikorodu in 2012 recruited 1,496 participants of all ages, which included 237 children under the age of five years and 509 children aged 15 years and below. Microscopy and DNA evaluation was used to determine parasite species, which found that 93.6% of participants had *P. falciparum* infection, with the remainder being *P. malariae* ^8^. ** This is in contrast to previous studies in 1976 around the same location in south-western Nigeria, which found that 13% to 16% of parasitaemia was due to *P. malariae*, with 62 to 76% being due to *P. falciparum* ^9^. These proportions were the age-group specific parasite prevalence in the study, which included mostly children aged five to ten years (1,500 participants) in an attempt to compare spleen and parasite rates among individuals with sickle cell trait and those with normal adult haemoglobin. It also found *P. ovale* in 2% to 3% of participants in 5–10 and 2–4 year age groups, respectively. These findings, although limited in scope, perhaps suggest a changing trend, with the disappearance of *P. ovale* from Nigeria over time. *P. falciparum* is an undisputed leader in all the studies performed. Its high proportion perhaps accounts for high rates of malaria-related anaemia in most studies, explained by the ability of the parasite to invade and destroy both young and senescent red blood cells ^8, 10^. Most of the data regarding the distribution of plasmodium is local and specific for locations where the studies are performed and the national estimates are mostly based on estimations. The knowledge needs to be updated constantly, in view of the changes over time, to help in appraising the efforts at controlling malaria.

We conducted this study to determine the true relative proportions of parasites causing clinical malaria in Sokoto, north-western Nigeria.

## Methods

### Ethical statement

Ethical approval was obtained from the Independent Ethics committee of Usmanu Danfoidyo University Teaching Hospital, Sokoto, Nigeria with ethical approval number UDUTH/HREC/2014/No. 246. Ethical approval was also obtained from the Independent Ethical committee of the Sokoto State Ministry of Health with the approval number SMH/1580/VIV. Permission was also obtained from the district heads of the included communities in the study to visit the communities.

### Study setting

The study was conducted in Wamakko Local Government Area of Sokoto State, located in the north-western geopolitical zone of Nigeria. It has an area of 732.146km ^2^, with a projected population of 260,860 by 2019 ^11^. It is located at coordinates 13°2′16″N 5°5′37″E. The geography of the area is predominantly flat plains with Sudan Savannah-type vegetation and it stands at an altitude of 292m above sea level, near to the confluence of the Sokoto and Rima rivers. Its climate is tropical, described as local steppe climate.

### Study design

The study was a two–point, cross-sectional prospective descriptive study conducted during the rainy season and dry season. In April and November 2016, we screened and recruited participants simultaneously until we reached the target population.

We determined the minimum sample size using Cochran’s formula ^12^, assuming a prevalence of 50% based on a previous survey ^13^, and 500 participants gave a power of at least 80% to show reliable results.

### Sampling technique

As is the norm for MIS’s, multistage cluster sampling in proportion to size was employed. The sample was stratified in two stages. First by the political wards and second by settlements. The samples were selected at each stratum independently. In the first stage, the strata were selected proportionate to their size. The details of the settlements are in the supplement number 1. The primary clusters were four randomly selected wards of the eleven political wards within the Local Government Area (LGA), with secondary clusters being eight settlements randomly selected from the four wards; proportionate to size, making up the total sample size. Based on the assumptions of a 70% response rate and that 80% of households include at least one child less than five years of age, in accordance with a previous Nigeria MIS in 2010 ^14^, approximately 892 households were required to meet the target of at least 500 participants per season based on the assumptions stated earlier. This was surpassed by the estimated number of households within the eight settlements selected. All children in the secondary clusters who fulfilled the inclusion criteria, and whose parents consented to participate, were included in the study.

### Sample population

Participants were visited at their homes and while in the house, after identifying the household head. They were provided with information regarding the study and all eligible children within the household invited to participate. Those who accepted to have the children in the household included in the study signed or thumb-printed the informed consent form. All children in the selected settlements who met the age criteria of two to 10 years, with or without symptoms of malaria, were recruited for the study, provided they had been residents of the study area for at least two weeks. They were, however, excluded if they were suspected to have taken any medication with antimalarial properties within the two weeks prior to enrolment ^36^. Recruitment was carried out on consecutive days until the entire village was covered. Each participant was evaluated once except for those who had parasitaemia without symptoms, who were followed up by a field assistant for up to 48 hours for the development of symptoms. The period of recruitment was about a month in each season.

### Procedures

We conducted the study procedures at a central location in each of the study villages. Field assistants went from house-to-house and recruited the participants and then brought the consenting participants to the central location. The lead investigator screened potential participants for eligibility and the caregivers of eligible participants were required to sign informed consent forms. All recruited subjects were issued with a unique study number, with which they were identified for the entire study. He performed a physical examination for each participant and graded splenomegaly according to Hackett’s criteria ^15^. The WHO criteria for severe malaria was used ^16^ to diagnose severe clinical malaria. Using a single use lancet, we collected capillary blood by pricking the index finger of the child’s left hand. A drop of blood was collected each for a thick and thin malaria parasite film for estimation of parasite density and species identification, respectively.

Concomitantly, we did rapid diagnosis of malaria using a drop of blood with CareStart® Malaria HRP2 rapid detection tests (RDTs) (Access Bio, Inc., model G0141), which can detect *P. falciparum*.

Diagnosis of malaria parasitaemia was done using malaria microscopy. Each day, we transported the samples to the paediatric department laboratory of the Usmanu Danfodiyo University Teaching Hospital and fixing of thick films was done with methanol. Thin films were stained immediately and stored in the lab. We analysed the samples in a completely anonymized manner in pairs of thick and thin films; examining the thin film if we found the thick film positive for malaria. The study numbers were the only identifiers for the thick films, which were kept apart from the thin films. A trained malaria microscopist performed the analysis, under the supervision of a medical parasitologist. We examined at least 10 fields before a slide was declared negative for malaria parasites.

The tail segment of the thin films was viewed by the lead investigator to identify the species of malaria parasite, using the typical description of parasite species, having been trained on parasite identification ^17^.

 Quality control of the diagnosis of the parasitaemia was provided by a trained medical microbiologist re-examining 10% of the slides selected at random. A discrepancy of 10% or more would have necessitated reanalysis of all the thick films and the thin films subsequently. The discrepancy was 3% (kappa score of 0.71) and as such, this was not necessary.

Study participants were determined to have clinical malaria, by the presence of at least one symptom of malaria and a positive RDT or thick blood film. These were treated by the study paediatrician at home with Artemether-Lumefantrine. Children were dosed according to standard dosing ^18^ but only the first dose was directly observed. Those with severe malaria were determined using the WHO criteria for severe malaria. These children were treated with an initial intramuscular dose of artesunate dosed according to the age ^19^ and referred immediately to the nearby tertiary hospital (UDUTH).

### Statistical analysis

We analysed the data using SPSS version 22. We determined the prevalence of malaria by parasitaemia and RDT by determining proportions no tested positive by each method/ no tested. We used descriptive statistics to determine averages and proportions. Participants with missing data were excluded from the analysis. We carried out sub-group analysis for age, gender and season and used kappa analysis to control the quality of malaria diagnosis.

## Results

### Participants included

**Figure 1.**
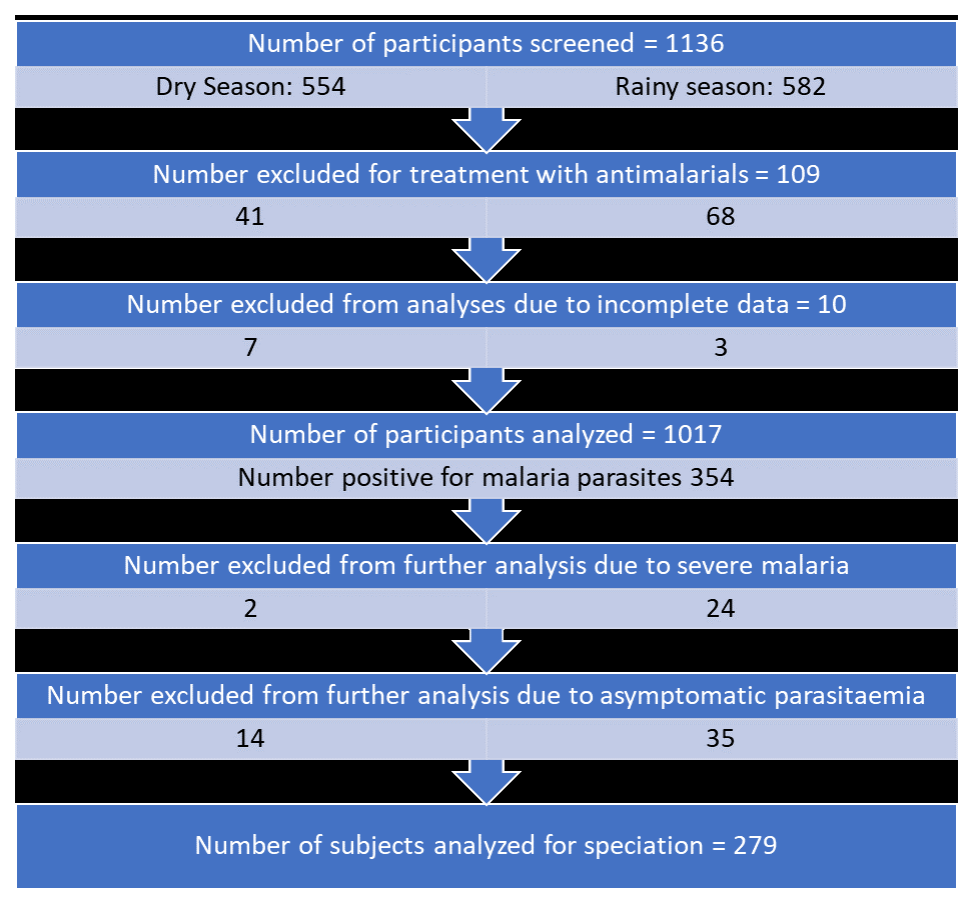
Flow chart for inclusion in analysis for clinical malaria.

We screened a total of 1136 participants for inclusion in the study after they consented to participation in the study. We excluded 109 because they had been treated with antimalarials in the two weeks prior to enrolment and excluded 10 from the analysis due to incomplete data. We included 1017 participants in the analysis ( Figure 1) ^20^.

The age-sex distribution showed that all ages were equally represented in the study, as shown in Table 1.

**Table 1.** Age and gender distribution of the subjects included in the study.

Age (completed years)	n	Gender	
		Male, n (%)	Female, n (%)
2	123	60 (5.9)	63 (6.2)
3	117	63 (6.2)	54 (5.3)
4	110	54 (5.3)	56 (5.5)
5	107	52 (5.1)	55 (5.4)
6	111	62 (6.1)	49 (4.8)
7	111	73 (7.2)	38 (3.7)
8	110	50 (4.9)	60 (5.9)
9	113	54 (5.3)	59 (5.8)
10	115	57 (5.6)	58 (5.7)
Total	1017	525 (51.6)	492 (48.4)

### Prevalence of malaria parasitaemia

We found an overall prevalence of malaria for the study of 34.8% using microscopy and 33.8% using RDT as shown in Table 2. There was an agreement between the two diagnostic methods, as shown by the kappa statistic (p <0.001).

**Table 2.** Prevalence of malaria parasitaemia among children aged 2-10 years using microscopy and rapid detection test (RDT).

Test result		Thick film		
		Positive	Negative	Total (%)
**RDT**	Positive	295	49	344 (33.8)
	Negative	59	614	673 (66.2)
	Total (%)	354 (34.8)	663 (65.2)	1017 (100.0)

Kappa agreement κ = 0.764; p <0.001.

### Age-specific prevalence rate of malaria parasitaemia

We saw the highest age-specific prevalence among participants aged two years, with the lowest among ten-year olds. Children aged three years of age at the time of the study had the highest frequency of uncomplicated clinical malaria (42.7%), while the highest frequency of severe complicated malaria was seen among 2-year-olds (9.8%). Children aged 10 years, had the lowest proportion of infected children showing complicated or uncomplicated clinical disease (66.7%) Table 3 also shows a significant association between the age of the participants and prevalence of malaria parasitaemia (p= 0.000).

**Table 3.** Age-specific prevalence of malaria parasitaemia and clinical malaria.

Age (completed years)	n	Malaria parasitaemia		Uncomplicated Clinical Malaria	Severe Malaria	Proportion of infected children with clinical disease
		Positive Freq (%)	Negative Freq (%)	Positive Freq (%)	Positive Freq (%)	%
2	123	62 (50.4)	61 (49.6)	46 (37.4)	12 (9.8)	93.5
3	117	57 (48.7)	60 (51.3)	50 (42.7)	4 (3.4)	94.7
4	110	37 (33.6)	68 (66.4)	30 (27.3)	2 (1.8)	86.5
5	107	39 (36.4)	68 (63.6)	30 28.0)	4 (3.7)	87.2
6	111	38 (34.2)	73 (65.8)	31 (27.9)	0	81.6
7	111	32 (28.8)	79 (71.2)	24 (21.6)	1 (0.9)	78.1
8	110	30 (27.3)	80 (72.7)	25 (22.7)	1 (0.9)	86.7
9	113	35 (31.0)	78 (69.0)	27 (23.9)	2 (1.8)	82.9
10	115	24 (20.9)	91 (79.1)	16 (13.9)	0	66.7
Total	1017	354 (34.8)	663 (65.2)	279 (27.4)	26	86.2

χ ^2^ = 38.453; df = 8; p<0.01.

### Parasite species causing malaria

**Figure 2.**
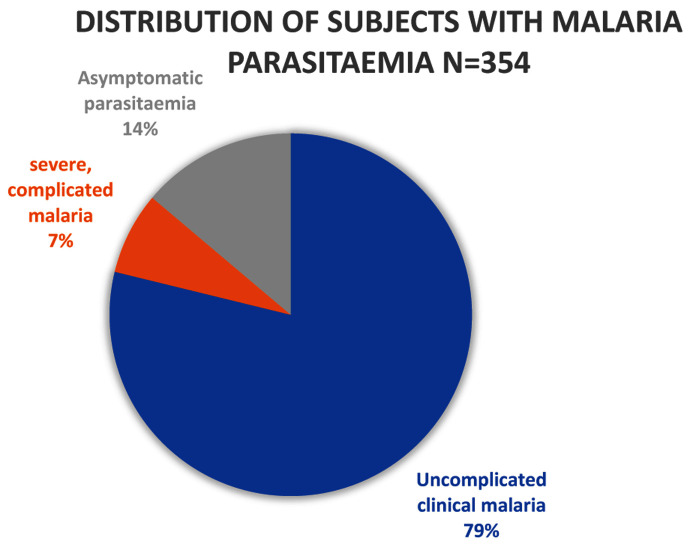
Distribution of nature of malaria infections.

Of the 354 children included in the study with malaria parasitaemia by microscopy, 279 participants were found to have clinical malaria across the seasons and all of them had *P. falciparum* malaria, irrespective of the season and nature of their clinical presentation. The relative proportions of the results of malaria infection are depicted in Figure 2. The figure basically shows the proportion of children with any malaria parasiatemia (34.8%) of which, 92% had fever. The most common presenting feature among these was fever (92%), followed by vomiting (38%), refusal to feed/poor appetite (32%) and body weakness (25%).

Aside the uncomplicated clinical cases of malaria, 9.6% had complicated malaria, as indicated by the WHO criteria for severity ^21^. The number of severe malaria cases was significantly lower in the dry season than the rainy season, as shown in Table 4. Different participants had various combinations of the criteria for severity, although the common criteria were hyperpyrexia, prostration and persistent vomiting.

**Table 4.** Prevalence of severe malaria across seasons.

Season	N	Severe malaria Freq (%)	Other cases Freq (%)
Rainy season	511	24 (4.7)	487 (95.3)
Dry season	506	2 (0.4)	504 (99.6)
Total	1017	26 (2.6)	991 (97.4)

χ ^2^ = 18.883; df = 1; p = 0.000.

### Comparison of prevalence between the seasons

The prevalence of malaria parasitaemia during the rainy season was significantly higher than the dry season, with prevalence rates of 49.3% and 20.2%, respectively, all due to *P. falciparum*.

### Parasite density across the seasons

The mean parasite density was much higher during the rainy season (1006.13) than during the dry season (405.45). The details are shown in Table 5.

**Table 5.** Parasite density across the seasons (parasites/µL).

Season	Mean	SD	Minimum	Maximum	Interquartile range
Rainy	1006.13	495.8	16	284000	840
Dry	405.45	209.48	16	204000	640
Overall	833.1	443.8	16	284000	1064

## Discussion

The prevalence of malaria in this study, when compared with serial MIS’s performed in 2010 ^14^ and 2015 ^22^ shows a progressive reduction; from 48.1% to 37.1% for north-western Nigeria, and a prevalence of 46.6% for Sokoto in 2015 compared with 34.8% for our study. Prevalence in the MIS’s was measured among children aged six to 59 months and is probably higher than for the children included in this study because including the children from 6–10 years is likely to reduce the overall prevalence, as is excluding those aged six months to two years, who generally have a higher prevalence rate ^23^. This suggests that the prevalence and intensity of malaria transmission has declined over time, particularly when it is juxtaposed against the rate of uptake of Long-Lasting Insecticide Treated Nets (LLINS) ^14, 22^.

 Although there were studies performed in the past in Sokoto, they are limited in comparison to the present study by virtue of having been conducted in a different age group, or hospital in lieu of community setting and the seasons in which these studies were conducted. The prevalence of 34.8% found here was higher than the 27.9% found by Abdullahi *et al.* ^13^ in Sokoto; however, samples in the previous study were collected from patients visiting two hospitals within the metropolis and was thus not community-based. Furthermore, all ages from 0 to 65 years were included in the study, which is likely to further dilute the findings and give a falsely low prevalence because the incidence of malaria is generally lower among adolescents and adults, as indicated in the study. Further lending credence to the claim of a reduction in the prevalence of malaria, likely owing to better access to malaria prevention and increasing urbanisation; both of which cause a decline in malaria parasite rates generally ^24^.

The prevalence found in this study is also lower in comparison to the 45.4% prevalence rate found in a study by Jiya *et al.* ^25^, conducted in Sokoto between 2007 and 2009. Additionally, it was lower than the prevalence of 49.6% found among children under the age of five years in the same study. Considering both age-specific prevalence rates, there is a reduction in prevalence, although being a hospital-based study, the prevalence for the former study is likely to be higher than the current. It is, however slightly, higher than the projected national average of 29% for 2015, with wide inter-regional differences ^26^. The Nigerian MIS of 2015 found a higher prevalence of 46.6% than this study, although the age of included participants ranged from six to 59 months, which will limit the comparability of results from this study due to the different age ranges of participants ^22^. This decline in the prevalence should reflect in the distribution of the parasites as theorised by Lucas and Gilles ^27^ who propounded a theory of disappearance of species as control measures improve until complete elimination is achieved. 

The prevalence by age in this study roughly indicated a progressive decline with age. The highest age-specific prevalence was among two-year-olds (50.4%), with a statistically significant difference among the age groups. This finding is in conformity with the steady-state assumption and is like findings in previous studies that showed higher prevalence among younger age-groups. The prevalence of uncomplicated clinical malaria was highest among three-year-olds and two-year-olds, and lowest among ten-ten-year olds, also in keeping with the steady-state-theory. Considered altogether, the under-fives constituted the largest proportion of uncomplicated and complicated malaria cases, and this is to be expected, considering that they are the least immune to malaria.

With respect to the parasite species causing uncomplicated malaria, all parasitaemia in this study was found to be due to *P. falciparum*. This is similar to findings in recent studies in Adamawa ^28^ and Cross River states ^10^ in 2011 and 2013, respectively. Another study from Ihiala, in Anambra state of south-eastern Nigeria, found *P. falciparum* mono-parasitaemia even though this study considered all types of malaria, both severe and uncomplicated ^29^. This is, however, unlikely to affect the findings, as most cases of severe malaria in this area are due to *P. falciparum* ^29^ *.* An earlier report from Sokoto between 2005 and 2006, carried out at Usmanu Danfodiyo University Teaching Hospital by Jiya and Sani ^25, 30^ likewise did not find any *Plasmodium* species apart from *P. falciparum*, although they only considered cases of severe malaria, which are unlikely to be due to a different species of *Plasmodium* within Nigeria. Meanwhile, only three cases out of 582 (0.01%) were positive for *P.malariae* in another study by Nwaorgu and Orajaka ^31^ in Awka, south-eastern Nigeria. A major limitation of the studies described is the population selected for hospital-based studies, which was mostly children with severe, complicated malaria, for which there is likely to be monoparasitaemia. The community-based studies are expected to truly reflect the distribution of parasite species because in the absence of radical-cure treatment with primaquine as is the case in Nigeria, *P. malariae and P. ovale* infections are likely to persist in the population. Other studies in the past had not explicitly described the process of identifying other parasite species and that could be a weakness in those studies. However, in our study which was community-based, we pound *P. falciparum* monoparasitaemia in spite of methodologically searching for other *Plasmodium* species. This leads us to believe that this is a true *P. falciparum* monoparasitaemia, contrary to expectation. The finding of *P. falciparum* mono-parasitaemia supports the likelihood that *P. falciparum* is the dominant species of *Plasmodium* in Sub-Saharan Africa, showing at tendency to exclude other forms of parasitaemia, as expounded by Lucas and Gilles ^27^ in 1998 with time and sustained malaria control activities.

 Other studies have expectedly shown the presence of other forms of parasitaemia, notably with *P. malariae* either as mono-infection or coinfection with *P. falciparum*. In Abia and Plateau states (2010), *P. malariae* accounted for 32.0% and 1.4% of malaria infections, respectively ^7^ in malariometric surveys In another community-based study in north-central Nigeria, 6.1% of examined participants had *P. malariae* infection ^32^ and as high as 41% and 4%, respectively, had *P. malariae* and *P. ovale* infections in a historical study in Garki, Abuja (1968) ^33^. Outside Nigeria, there has also been a documented shift towards mono-parasitaemia with *P. falciparum*, and this has been documented in the Horn of Africa ^34^ and Benin in West Africa ^35^. Some authors have suggested it be an evidence of failing control measures but this is at variance with data from this study, which shows a reduction in prevalence from previous data, including a reduction in the number of severe cases of malaria, which were very few in this study both in the rainy and dry seasons, reflecting better malaria control than seen in previous studies.

Severe malaria was seen in 26 of the 1017 participants analysed in this study, with an overall prevalence of 2.6%. It was higher during the rainy than the dry season, probably due to higher prevalence of the disease and higher parasitaemia, as earlier discussed. This is lower than expected from other hospital-based studies for which children presenting to the hospital are more likely to be ill than those found in a community-based survey such as this. In one such study in Ilorin by Olanrewaju and Johnson ^27^ found that a third of all children admitted with malaria had a severe form of malaria.

Uncomplicated, clinical malaria was seen in 279 of the children in the study (27.4%), which is relatively high, considering that it was community based. The proportion of tested children with malaria parasitaemia who had uncomplicated malaria was also high overall (86.2%) and higher in the rainy than the dry season. The role of Seasonal Malaria Chemoprophylaxis (SMC) would thus be relevant in reducing this percentage of positives with clinical malaria ^36^.

Our study is limited by the point estimation of malaria prevalence, for which a time-series would have been preferred to document consistently the changes over time. Also, the use of RDT kits, could have been complimentary to microscopy for the diagnosis of malaria species if we used a kit with the ability to detect other parasite species.

## Conclusions

We found that the area had an intermediate endemicity of malaria transmission. Despite this, there was *P. falciparum* monoparasitaemia. This suggests a disappearance of other parasite species, either due to faltering control of *P. falciparum* leading to dominance by these species or more efficient control of other species. However, the trend of overall endemicity suggests the latter explanation, implying a need to intensify measures to control *P. falciparum*, considering its strategic role in causing clinically severe malaria in SSA.

## Data availability

### Underlying data

Figshare: complete data.xlsx. https://doi.org/10.6084/m9.figshare.11590542.v1 ^20^.

Data are available under the terms of the Creative Commons Attribution 4.0 International license (CC-BY 4.0).
