# Supplementary material for: Where have all the parasites gone? Unusual Plasmodium falciparum monoparasitaemia in a cross-sectional malariometric survey in northern Nigeria
Source: F1000Res. 2020 Oct 27;9:301. Originally published 2020 Apr 28. [Version 2] doi: 10.12688/f1000research.20997.2 (PMC7658729; doi:10.12688/f1000research.20997.2)
Supplement: Peer review history

# Peer review: Reviewer response for version 2

**DOI:** 10.5256/f1000research.29448.r73706

**Published:** 2020-11-20

**Authors:** Manuela Runge, Ifeoma Ozodiegwu

**Affiliations:** 1 Northwestern University, Evanston, IL, USA

**Major Comments:**

- The title and results section still seems disconnected and not present what one would expect from the title. The monoparasitameia result is only mentioned in ‘Comparison of prevalence between the seasons’. The result section mostly describes malaria parasitaemia and severity by age and season.
- The paragraph heading “Parasite species causing malaria” does not include parasite species and the author might want to change the subheading to match the text.
- Comparing observed prevalence in the study with other studies is inappropriate because of seasonality. The authors should have used the discussion to discuss what monparastemia means for the control and elimination in Sokoto. What interventions are ongoing and how can program planners and policy makers use their findings?

**Minor Comments:**

- The reported prevalence estimates throughout the results section are based on microscopy or mRDT (for example in Comparison of prevalence between the seasons). Maybe the authors could add a simple sentence in the methodologies that all prevalence are from microscopy if not otherwise stated?
- Table 4 still shows only severe malaria, although the objective of the study was described with a focus on uncomplicated.
- Abstract result “Mapping of the parasite was extended throughout the Local Government Area and the State” is not very clear, consider rephrasing “Mapping of the parasite”.
- In the section “Parasite density across the seasons” the units for the parasite density numbers are missing.
- Under “Age-specific prevalence rate of malaria parasitaemia” significance value (p=0.000) was changed from previously (p=<0.01), which seemed better practice, why the change?

Is the work clearly and accurately presented and does it cite the current literature?

Partly

If applicable, is the statistical analysis and its interpretation appropriate?

Yes

Are all the source data underlying the results available to ensure full reproducibility?

Partly

Is the study design appropriate and is the work technically sound?

Partly

Are the conclusions drawn adequately supported by the results?

Yes

Are sufficient details of methods and analysis provided to allow replication by others?

Partly

Reviewer Expertise:

malaria epidemiology in school children, malaria surveillance, mathematical modelling of malaria

We confirm that we have read this submission and believe that we have an appropriate level of expertise to confirm that it is of an acceptable scientific standard, however we have significant reservations, as outlined above.

# Peer review: Reviewer response for version 2

**DOI:** 10.5256/f1000research.29448.r73707

**Published:** 2020-11-13

**Authors:** Linda Eva Amoah

**Affiliations:** 1 Immunology Department, Noguchi Memorial Institute for Medical Research, University of Ghana, Accra, Ghana

Most of my previous comments were addressed but some were not.

**Major concern**

Abstract

 The abstract states "...causing uncomplicated malaria....". This study should have enrolled only people with uncomplicated malaria. This sentence must be revised to reflect the total samples used.

**Minor concerns**

Results

 I still do not understand why some malaria indices were stratified by age and also by season and others including RDT positivity rate was not.

Discussion

 "the prevalence" revise to state what prevalence you are referring to.

Is the work clearly and accurately presented and does it cite the current literature?

Partly

If applicable, is the statistical analysis and its interpretation appropriate?

Partly

Are all the source data underlying the results available to ensure full reproducibility?

Yes

Is the study design appropriate and is the work technically sound?

Yes

Are the conclusions drawn adequately supported by the results?

Partly

Are sufficient details of methods and analysis provided to allow replication by others?

Yes

Reviewer Expertise:

Recombinant DNA technology and Malaria transmission, Plasmodium species identification, gametocytes and serology as well as host genetics

I confirm that I have read this submission and believe that I have an appropriate level of expertise to confirm that it is of an acceptable scientific standard, however I have significant reservations, as outlined above.

# Peer review: Reviewer response for version 2

**DOI:** 10.5256/f1000research.29448.r73708

**Published:** 2020-11-11

**Authors:** Richard Mwaiswelo

**Affiliations:** 1 Department of Microbiology, Immunology and Parasitology, Hubert Kairuki Memorial University, Dar es Salaam, Tanzania; 2 Department of Parasitology and Medical Entomology, Muhimbili University of Health and Allied Sciences, Dar es Salaam, Tanzania

I approve the manuscript to be indexed, however, I have some minor comments:

1. "Malaria is caused by one of five currently known *Plasmodium* species causing diseases in humans." - Rephrase the sentence to read, "In Nigeria, malaria is caused predominantly by one of the five currently……………in humans."
2. *"P. falciparum* is an undisputed leader in all the studies performed." - Rephrase the sentence to read, " *P. falciparum* is an undisputed predominant parasite in all the studies performed."
3. "Its high proportion perhaps accounts for high rates of malaria-related anaemia in most studies, explained by the ability of the parasite to invade and destroy both young and senescent red blood cells ^8^ ^,^ ^10^." - This sentence is redundant and I suggest for it to be removed.

Is the work clearly and accurately presented and does it cite the current literature?

Partly

If applicable, is the statistical analysis and its interpretation appropriate?

Partly

Are all the source data underlying the results available to ensure full reproducibility?

Yes

Is the study design appropriate and is the work technically sound?

Yes

Are the conclusions drawn adequately supported by the results?

No

Are sufficient details of methods and analysis provided to allow replication by others?

Partly

Reviewer Expertise:

Malaria

I confirm that I have read this submission and believe that I have an appropriate level of expertise to confirm that it is of an acceptable scientific standard.

# Peer review: Reviewer response for version 1

**DOI:** 10.5256/f1000research.23106.r69663

**Published:** 2020-09-18

**Authors:** Linda Eva Amoah

**Affiliations:** 1 Immunology Department, Noguchi Memorial Institute for Medical Research, University of Ghana, Accra, Ghana

**Abstract/Introduction:**

1. What is monoparasitaemia? Do you mean mono species parasitaemia?
2. The first sentence of the abstract/introduction should be revised as malaria in other animals is caused by other *Plasmodium *species.
3. The reference WHO 2014 and 2016 are not current, what is the current data?
4. What is meant by ‘parasite mapping’ and why is this important to the reader?
5. Check the text and remove spaces before full stops as well include a space before the falciparum in *P. falciparum*.
6. ‘not so much the parasite’? I do not understand what this means. There is no modeling in this study so the second sentence in the introduction is not relevant, especially as it is very confusing.
7. What is the definition of ‘confirmed cases?’
8. What does ‘crucially’ mean?

**Methods:**

1. Is April the peak of the malaria season? The description of malaria indices in the study site is not sufficient.
2. The blood draw procedure can be rewritten to enhance clarity. Overall, how many drops of blood were collected? And what processes/procedures were performed?

**Tables:**

1. Tables 1, 2, 3, and 5 can easily be merged. It would reduce the repetition.
2. Table 2 can be stratified by age as the other tables.
3. Table 4 should be age stratified and other cases separated into asymptomatic and uncomplicated cases.

**Figure 2: **

1. The title is wrong as it includes ‘no parasites’. No parasites do not cause malaria.
2. There are items in the legend that are not on the graph, it would be best to remove and just state in the foot notes that none of those other species were identified.

**Results:**

1. Was the parasite density at the two time points significantly different?

**Discussion:**

1. It would be very easy to compare the results from the current study with the data in the MIS by comparing similarly aged children. The statement can be revised if the analysis is redone for children aged 5 years and below.
2. I think the last paragraph should be a summary of the major findings and provide information related to the main aim or relevance to the title but it is presently not so.

Is the work clearly and accurately presented and does it cite the current literature?

Partly

If applicable, is the statistical analysis and its interpretation appropriate?

Partly

Are all the source data underlying the results available to ensure full reproducibility?

Yes

Is the study design appropriate and is the work technically sound?

Yes

Are the conclusions drawn adequately supported by the results?

Partly

Are sufficient details of methods and analysis provided to allow replication by others?

Yes

Reviewer Expertise:

Recombinant DNA technology and Malaria transmission, Plasmodium species identification, gametocytes and serology as well as host genetics

I confirm that I have read this submission and believe that I have an appropriate level of expertise to confirm that it is of an acceptable scientific standard, however I have significant reservations, as outlined above.

# Response

**Published:** 2020-09-19

**Authors:** Usman Nasir Nakakana

**Affiliations:** MRC The Gambia Unit at LSHTM, The Gambia

**Abstract/Introduction:**

1. What is monoparasitaemia? Do you mean mono species parasitaemia?
**Yes, this is clarified in the text**
2. The first sentence of the abstract/introduction should be revised as malaria in other animals is caused by other *Plasmodium *species.
**It has been rephrased for clarity**
3. The reference WHO 2014 and 2016 are not current, what is the current data? **The data was collected in 2016 and written up in 2017, the data is referencing the time around which the data was collected to ensure comparability, considering the constantly changing data**
4. What is meant by ‘parasite mapping’ and why is this important to the reader? **Parasite mapping refers to the description of geographical spread of the prasite species and how it can be extended to other parts of the state, based on climatic and geographic information.**
5. Check the text and remove spaces before full stops as well include a space before the falciparum in *P. falciparum*.
**Checked and corrected**
6. ‘not so much the parasite’? I do not understand what this means. There is no modeling in this study so the second sentence in the introduction is not relevant, especially as it is very confusing.
**Reference to modelling is deleted, the statement has been rephrased**
7. What is the definition of ‘confirmed cases?’ confirmed cases is defined as those with a positive malaria parasite test and clinical symptoms.
**This has been elaborated**
8. What does ‘crucially’ mean?
**Changed to 'notably'**

**Methods:**

1. Is April the peak of the malaria season? The description of malaria indices in the study site is not sufficient.
**Some information has been provided**
2. The blood draw procedure can be rewritten to enhance clarity. Overall, how many drops of blood were collected? And what processes/procedures were performed?
**It has been re-written to increase clarity**

**Tables:**

1. Tables 1, 2, 3, and 5 can easily be merged. It would reduce the repetition. **Table 3 contains more data including data on the age distribution**
2. Table 2 can be stratified by age as the other tables.
**The data is provided in table 3**
3. Table 4 should be age stratified and other cases separated into asymptomatic and uncomplicated cases.
**The data is provided in table 3**

**Figure 2: **

1. The title is wrong as it includes ‘no parasites’. No parasites do not cause malaria.
2. There are items in the legend that are not on the graph, it would be best to remove and just state in the foot notes that none of those other species were identified.
**The figure has been changed entirely in version 2**

** Results:**

1. Was the parasite density at the two time points significantly different? **There was no test of significance performed but it is much higher in the rainy than the dry season.**

**Discussion:**

1. It would be very easy to compare the results from the current study with the data in the MIS by comparing similarly aged children. The statement can be revised if the analysis is redone for children aged 5 years and below.
**The prevalence of malaria is described as it relates to the parasite species distribution and the impact of malaria endemicity. The metric for endemicity is the prevalence of falciparum malaria among children 2 to 10 years of age, which is the age group we studied. We also did not collect any data in children 6 months to 2 years and so even if we consider children under 5 years, we still would not be able to compare the data directly with MIS data.**
2. I think the last paragraph should be a summary of the major findings and provide information related to the main aim or relevance to the title but it is presently not so.
**It has been revised**

# Peer review: Reviewer response for version 1

**DOI:** 10.5256/f1000research.23106.r69658

**Published:** 2020-09-15

**Authors:** Richard Mwaiswelo

**Affiliations:** 1 Department of Microbiology, Immunology and Parasitology, Hubert Kairuki Memorial University, Dar es Salaam, Tanzania; 2 Department of Parasitology and Medical Entomology, Muhimbili University of Health and Allied Sciences, Dar es Salaam, Tanzania

This article presents findings from a cross-sectional study conducted in Wamakko Local Government Authority, in Sokoto State in Nigeria with a broad objective of determining the prevalence of *Plasmodium *parasite species causing uncomplicated malaria in human in the area. The diagnosis of malaria was performed using mRDTs (for the detection of the presence of infection) and thick (for assessing parasite density) and thin (for species identification) smears. The key finding is *Plasmodium falciparum* monoinfection in the study area. 

**Major Comment**

 The authors have failed to answer the following questions so as to convince readers, and thus should be addressed: What is the aim of this study? Why do you want to understand the prevalence of different malaria parasite species present in that part of Nigeria? How does the understanding of the prevalence of the parasite species contribute to the management, control and prevention of malaria in general? Try to explain this so as to lay down the significance of this study.         

**Abstract**

***Background***

- The first sentence…………'Malaria is caused by one of five currently known Plasmodium parasite species’…the sentence is not clear. Where do the authors refer to where malaria is caused by that one of the five species?
- What information on vector does the modeling provide? The sentence is redundant; I suggest to be omitted. 
- The sentence………’This study aimed to determine proportions of Plasmodium parasites’………is not in line with the true aim of the study. It would sound clear if it was stated that the aim was to determine the proportions of *Plasmodium* species infecting humans in the study area.

***Methods***

- ‘Selected participants were examined for ………. signs of complicated malaria'. However, there is nowhere in the abstract were it has been stated that this was either a hospital or community based study, and whether only symptomatic or both symptomatic and asymptomatic individuals for malaria infection were included in the study. How were the study participants selected, randomly or conveniently? What was the age limit of the study participants? 
- ‘…………and thick and thin films to identify parasitemia and parasite species’. Which one of the tests was used to identify parasitemia, and which one was used to identify species? Be specific.
- ‘………. with complicated malaria were referred to the nearest hospital’………but you are silent about those individuals with uncomplicated malaria, were they treated?

***Results***

- ‘There was monoparasitemia of Plasmodium falciparum’…………. I suggest to rewrite the sentence and read……………. There was Plasmodium falciparum monoinfection throughout…….
- On the other hand, detection of parasite species was based on microscopy thin smears, where there any efforts to detect the parasite species using more sensitive methods such as PCR?
- ‘Mapping of the parasite…….’This sentence is not part of the results but rather of the methods, move it to the methods section.

***Conclusion***

- Omit the sentence……………...’Despite the intermediate endemicity in the area’…………. otherwise, how does the endemicity determine the presence of a certain malaria parasite specie? 

 

**Introduction**

- The first sentence is confusing, same as in the abstract. See the above comment.
- The third sentence………...’scientists have modelled the Anopheles………...but not so much on the parasite’. This sentence is misleading, and which characteristics are you referring to? The sentence itself is redundant and I suggest to be omitted.
- This figure likely overestimated the proportion of cases………...tracked by passive surveillance in Nigeria’. The sentence is not clear, rephrase it to increase clarity.
- These proportions were the age-group specific parasite prevalence……………...sickle cell trait and those with normal adult hemoglobin. Rephrase this sentence to increase clarity.
- ‘Plasmodium falciparum is an undisputed leader’……………. I don’t think the word leader suits well in this sentence, I suggest the use of the word ‘predominant/prevalent’.
- There is no aim of the study in the whole introductory section.

**Methods**

***Study settings***

- How many rainfall seasons are there in the study area? When does the rainfall season start and end? What are the major economic activities in the area? What are the major vectors? What are the major malaria control tools employed in the area?

***Study design***

- Where the participants selected randomly or conveniently?
- Was this a community-based or health facility-based study?

***Sample population***

- Rewrite sentences 1 and 2 to increase clarity……...’after identifying the household head. They were provided with information regarding the study’………. To who was the information provided, the household head, or everyone in the household? Who gave the consent?
- ‘Residents of the study area for at least two weeks’…………...with this very short time of being a residency in the study area, how sure are you that the malaria parasite species found in the assessed individuals have not originated out of your study area (where the assessed individual was living before moving into the study community)?
- Except for those who had parasitemia without symptoms……...followed up………. for up to 48 hours for the development of symptoms. Is it ethical to not treat an infected individual and only wait for symptoms to occur?

***Procedures***

- Does this mean that the field assistant recruited the study participants prior to obtaining consent from parents/guardians? Is this ethical?
- ‘We examined at least 10 fields before a slide was declared negative’……….what is the standard number of fields that are required to be read before declaring a slide negative?

***Statistical analysis***

- ‘We determined the prevalence of malaria by parasitemia and RDT by determining proportions'. The sentence is not clear, rewrite it.

**Results**

- Combine tables 1 and 3
- Combine tables 4 and 5
- Figure 2………..the prevalence of other parasite species is 0%, I suggest you find another method for presenting this, otherwise the figure is not convincing. 
- The following sub-sections can be combined: Prevalence of malaria, age-specific and sex-specific prevalence of malaria, the prevalence of malaria by seasons, and parasite density by seasons. All these can be under one sub-section ‘Malaria prevalence’.
- You should have at least 3 sub-sections to include: Included participants, Prevalence of malaria, and Plasmodium parasite species.

**Parasite density across the season**

- ‘…………density was much higher during rainy…………., but the p-value is not given and the authors states that details are shown in Table 5, this is not proper.

**Discussion**

- Evaluation of the prevalence of malaria in the study area was not the main objective of the study, but rather the evaluation of the prevalence of *Plasmodium *species infecting humans in the study area was the main objective. Why then the first paragraph concentrate on the prevalence of malaria rather than of the infecting parasite species?
- The second sentence in the first paragraph is not clear, and what are you trying to suggest by ……...’as is excluding those aged 6 months to 2 years? How is malaria prevalence stratified by age, I mean what is the standard?
- The discussion has concentrated much on malaria prevalence rather than on the main objective of the study, which is the prevalence of Plasmodium species infecting humans in the study area.
- The conclusion is missing.

**References**

- Reference 2. World Health Organization…………. Nigeria. Geneva, 2014. Remove the name Nigeria.

Is the work clearly and accurately presented and does it cite the current literature?

Partly

If applicable, is the statistical analysis and its interpretation appropriate?

Partly

Are all the source data underlying the results available to ensure full reproducibility?

Yes

Is the study design appropriate and is the work technically sound?

Yes

Are the conclusions drawn adequately supported by the results?

No

Are sufficient details of methods and analysis provided to allow replication by others?

Partly

Reviewer Expertise:

Malaria

I confirm that I have read this submission and believe that I have an appropriate level of expertise to confirm that it is of an acceptable scientific standard, however I have significant reservations, as outlined above.

# Peer review: Reviewer response for version 1

**DOI:** 10.5256/f1000research.23106.r69662

**Published:** 2020-09-01

**Authors:** Manuela Runge, Ifeoma Ozodiegwu

**Affiliations:** 1 Northwestern University, Evanston, IL, USA

The paper presents findings from a cross-sectional study conducted in one LGA (Wamakko) in Sokoto State in Nigeria that aimed to determine the proportions of *plasmodium* parasites causing uncomplicated malaria. The study included data collection in both, rainy as well as the dry season. The diagnosis of malaria in study participants was done via rapid tests and dried blood spots, which in the study closely aligned with each other (3% difference). The key finding is monoparasitaemia in the study population with all of the malaria infections caused by *plasmodium falciparum*.

 However, it is not exactly clear why this study was performed. The authors did not make a clear statement of the gap in the literature or how their study complements the literature. The studies that they cited suggests that the monoparasitemia as it relates to malaria in Nigeria is a known fact. Is their goal to confirm this for a different geographical location? A more clearly written aims and study motivation will convince readers of the importance of their study

**Major comments**

1. Please elaborate on your methods.
  - If cluster sampling proportionate to size was used, list all the wards and settlement in a table with their population sizes. This can be included in the supplement.
  - How was cluster selection done? Did you stratify by ward and settlement sizes before random sampling? Be explicit about what was done and how it was done.
2. Please use the discussion section to discuss the implications of your study for treatment and control and the wider scientific community. What are the study limitations? The bulk of this section should not be spent comparing your findings with other studies. We would refer the authors to this link on how to write a discussion section. There are other resources online as well.
3. In the discussion, studies are cited that report monoparasitaemia, which seems circular/contradictory to the title of ‘ *unusual*’ monoparasitaemia, as the discussion indicates that this could have been expected?  The authors may consider rephrasing the title or to add a stronger argumentation in the introduction and discussion (see major comment 1).
4. The conclusions are missing in the main text, but included in the abstract.
5. The potential reasons for the disappearance “ *either due to faltering control of P. falciparum or more efficient control of other species*” (last part of the abstract conclusion) could be discussed more in the discussion as one would expect from the title.
6. Severe versus clinical malaria. The study aim states a focus on uncomplicated malaria, however, Table 4 only shows severe malaria, which should also show uncomplicated malaria (or instead). The WHO criteria for severe malaria were used, does that include criteria for uncomplicated malaria if not which criteria were used? (Paragraph on Procedures, line 9). How were severe cases treated in the study did they also receive ALu at home or were referred to the next hospital or health facility? Moreover, it would be relevant to show uncomplicated malaria per age in addition to parasitaemia in Table 3.

 

**Minor comments**

1. Previous studies reported the highest parasitaemia among school-aged children ^1^ and prevalence curves that peak among school children while incidence curves peak in children under the age of five ^2^. The article here found that parasitaemia is highest at age two and then decreases by age. A sentence for clarification on this would be helpful that explain the decrease over age?
2. The authors found 65% to be non-malaria fevers, this is interesting and the authors may consider highlighting it as a finding and adding it to the discussion (Fig 2).
3. “ *Scientists have modelled the anopheles mosquito vector extensively based on its characteristics, but not so much the parasite*” the reference to this study and to niche models of vectors is confusing, as the author’s study is not doing niche modelling or geographic mapping of parasites. It would seem more appropriate to have a description of the geographical occurrence of parasites leading over to the next sentence.
4. Missing reference for the Duffy antigen sentence line 6 to 9 in the first paragraph (“ *An absence of the Duffy antigen on red blood cells of West Africans has long been postulated to be responsible for the absence of P. vivax in these areas, but cases of P. falciparum, P. malariae and P. ovale, in order of decreasing occurrence, have been found*”).
5. “ *Participants were visited at their homes and while in the household*” Perhaps should be “while being at home”? Also, were participants visited after identifying the head, or happened both at the same time, visiting the household and then identifying the household head?
6. Confusing use of household head – parent - caregivers, are these refer all to the same (i.e. are household heads always parents?)
7. The authors cite the Nigerian national treatment policies for parasite co-infections, which however is not the main source which remains unclear, is there an actual study that could be cited?
8. “ *five to 10 years*” write out numbers below twenty, or use numbers consistently.
9. Possible gender bias in writing ‘he’ for the pediatrician “He performed a physical examination for each participant and graded splenomegaly according to Hackett’s criteria”.
10.  “ *Concomitantly, during the same session…”* consider removing duplication?
11. “ *The study numbers*” is not explained before; do the authors mean participant identifiers or study participant numbers?
12. Unclear sentence: “ *The tail segment of the thin films was viewed to identify the species of malaria parasite, using the typical description of parasite species, having been trained on parasite identification*”, who has been trained?
13. Unclear sentence: “ *We determined the prevalence of malaria by parasitaemia and RDT by determining proportions*.” Maybe the authors could add formula pos mRDT/tested by mRDt; pos BS/ tested by BS to be clearer?
14. Consider reducing the black background in figures and tables and not using 3D plots, see some general rules on scientific figures in ^3^.
15. Be consistent with ‘malaria’ or ‘clinical malaria’ to distinguish malaria infection from clinical malaria, i.e. Fig 2 caption and title, and text description.
16. Confusing Fig 2, as it reads as if ‘no parasite can also cause malaria’? Would a barchart be more appropriate? Or consider rephrasing the figure title.
17. Reference number 36 is missing or not needed (sample procedures line 11), reference list goes only up to 33.
18. ‘ *The finding of P. falciparum mono-parasitaemia supports the fact that P. falciparum is the dominant species of Plasmodium in Sub- Saharan Africa’ *Consider rephrasing like ‘confirms/ support the other findings of mono-parasitaemia’ to avoid the term ‘fact’.
19. The discussion structure might need some revision for better understanding, for example, the last paragraph on severe malaria in the discussion might fit better after discussing uncomplicated malaria and before discussing monoparasitaemia in other studies.

Is the work clearly and accurately presented and does it cite the current literature?

Partly

If applicable, is the statistical analysis and its interpretation appropriate?

Yes

Are all the source data underlying the results available to ensure full reproducibility?

Partly

Is the study design appropriate and is the work technically sound?

Partly

Are the conclusions drawn adequately supported by the results?

Yes

Are sufficient details of methods and analysis provided to allow replication by others?

Partly

Reviewer Expertise:

malaria epidemiology in school children, malaria surveillance, mathematical modelling of malaria

We confirm that we have read this submission and believe that we have an appropriate level of expertise to confirm that it is of an acceptable scientific standard, however we have significant reservations, as outlined above.

## References

[1] : Asymptomatic Plasmodium infection and cognition among primary schoolchildren in a high malaria transmission setting in Uganda. *Am J Trop Med Hyg*.2013;88(6) : 10.4269/ajtmh.12-0633 1102-1108 10.4269/ajtmh.12-0633 23589533PMC3752809

[2] : Estimates of the changing age-burden of Plasmodium falciparum malaria disease in sub-Saharan Africa. *Nat Commun*.2014;5: 10.1038/ncomms4136 3136 10.1038/ncomms4136 24518518PMC3923296

[3] : Ten Simple Rules for Better Figures. *PLoS Computational Biology*.2014;10(9) : 10.1371/journal.pcbi.1003833 10.1371/journal.pcbi.1003833 PMC416129525210732

# Response

**Published:** 2020-09-16

**Authors:** Usman Nasir Nakakana

**Affiliations:** MRC The Gambia Unit at LSHTM, The Gambia

1. Please elaborate on your methods.
  - If cluster sampling proportionate to size was used, list all the wards and settlement in a table with their population sizes. This can be included in the supplement.
  - How was cluster selection done? Did you stratify by ward and settlement sizes before random sampling? Be explicit about what was done and how it was done
  - **This has been done, there were two strata, selected independently. The first stratum was proportional to size, i.e at the ward level.**
2. Please use the discussion section to discuss the implications of your study for treatment and control and the wider scientific community. What are the study limitations? The bulk of this section should not be spent comparing your findings with other studies. We would refer the authors to this link on how to write a discussion section. There are other resources online as well.
**I have added sections and modified the discussion as per your recommendations. Implications to the wider scientific community are included.**
3. In the discussion, studies are cited that report monoparasitaemia, which seems circular/contradictory to the title of ‘ *unusual*’ monoparasitaemia, as the discussion indicates that this could have been expected?  The authors may consider rephrasing the title or to add a stronger argumentation in the introduction and discussion (see major comment 1).
**It has been explained why the results are unusual, given the population studied i.e community-based. this is reflected in the introduction and conclusions as suggested**
4. The conclusions are missing in the main text, but included in the abstract.
**They have now been included in the main text.**
5. The potential reasons for the disappearance “ *either due to faltering control of P. falciparum or more efficient control of other species*” (last part of the abstract conclusion) could be discussed more in the discussion as one would expect from the title.
**This has been included in the discussion.**
6. Severe versus clinical malaria. The study aim states a focus on uncomplicated malaria, however, Table 4 only shows severe malaria, which should also show uncomplicated malaria (or instead). The WHO criteria for severe malaria were used, does that include criteria for uncomplicated malaria if not which criteria were used? (Paragraph on Procedures, line 9). How were severe cases treated in the study did they also receive ALu at home or were referred to the next hospital or health facility? Moreover, it would be relevant to show uncomplicated malaria per age in addition to parasitaemia in Table 3.
**Table 3 has been modified to include other parameters such as the age-specific prevalence of uncomplicated and severe, complicated malaria. Figure 2 has also been modified for more clarity**

 

**Minor comments**

1. Previous studies reported the highest parasitaemia among school-aged children ^1^ and prevalence curves that peak among school children while incidence curves peak in children under the age of five ^2^. The article here found that parasitaemia is highest at age two and then decreases by age. A sentence for clarification on this would be helpful that explain the decrease over age?
**This has been explained as the steady-state assumption.**
2. The authors found 65% to be non-malaria fevers, this is interesting and the authors may consider highlighting it as a finding and adding it to the discussion (Fig 2).
**The figure has been changed for clarity. What is reported was that 34.8% of all children had malaria parasitaemia and the nature of parasitaemia i.e symptomatic and asymptomatic has been clarified.**
3. “ *Scientists have modelled the anopheles mosquito vector extensively based on its characteristics, but not so much the parasite*” the reference to this study and to niche models of vectors is confusing, as the author’s study is not doing niche modelling or geographic mapping of parasites. It would seem more appropriate to have a description of the geographical occurrence of parasites leading over to the next sentence.
**The sentence has been rephrased.**
4. Missing reference for the Duffy antigen sentence line 6 to 9 in the first paragraph (“ *An absence of the Duffy antigen on red blood cells of West Africans has long been postulated to be responsible for the absence of P. vivax in these areas, but cases of P. falciparum, P. malariae and P. ovale, in order of decreasing occurrence, have been found*”).
**Reference provided.**
5. “ *Participants were visited at their homes and while in the household*” Perhaps should be “while being at home”? Also, were participants visited after identifying the head, or happened both at the same time, visiting the household and then identifying the household head?
**Clarified that it happened in the house.**
6. Confusing use of household head – parent - caregivers, are these refer all to the same (i.e. are household heads always parents?)
**Clarified, the household head gives permission and it could be a parent or other person.**
7. The authors cite the Nigerian national treatment policies for parasite co-infections, which however is not the main source which remains unclear, is there an actual study that could be cited?
**The treatment guidelines are the ones cited.**
8. “ *five to 10 years*” write out numbers below twenty, or use numbers consistently.
**I thought this was one to nine, it has been edited.**
9. Possible gender bias in writing ‘he’ for the pediatrician “He performed a physical examination for each participant and graded splenomegaly according to Hackett’s criteria”.
**Clarified, the lead investigator is the corresponding author and is male.**
10.  “ *Concomitantly, during the same session…”* consider removing duplication?
**Done.**
11. “ *The study numbers*” is not explained before; do the authors mean participant identifiers or study participant numbers?
**Study number is explained.**
12. Unclear sentence: “ *The tail segment of the thin films was viewed to identify the species of malaria parasite, using the typical description of parasite species, having been trained on parasite identification*”, who has been trained?
**Clarified.**
13. Unclear sentence: “ *We determined the prevalence of malaria by parasitaemia and RDT by determining proportions*.” Maybe the authors could add formula pos mRDT/tested by mRDt; pos BS/ tested by BS to be clearer?
**Formula provided for each instance.**
14. Consider reducing the black background in figures and tables and not using 3D plots, see some general rules on scientific figures in ^3^.
**Figure changed.**
15. Be consistent with ‘malaria’ or ‘clinical malaria’ to distinguish malaria infection from clinical malaria, i.e. Fig 2 caption and title, and text description.
**Figure 2 has been modified.**
16. Confusing Fig 2, as it reads as if ‘no parasite can also cause malaria’? Would a barchart be more appropriate? Or consider rephrasing the figure title.
**Figure 2 changed.**
17. Reference number 36 is missing or not needed (sample procedures line 11), reference list goes only up to 33.
**References updated.**
18. ‘ *The finding of P. falciparum mono-parasitaemia supports the fact that P. falciparum is the dominant species of Plasmodium in Sub- Saharan Africa’ *Consider rephrasing like ‘confirms/ support the other findings of mono-parasitaemia’ to avoid the term ‘fact’.
**Fact has been deleted**
19. The discussion structure might need some revision for better understanding, for example, the last paragraph on severe malaria in the discussion might fit better after discussing uncomplicated malaria and before discussing monoparasitaemia in other studies
20. **Some modification has been done**
